# Methylene blue-assisted technique for harvesting lymph nodes after radical surgery for gastric cancer: a prospective randomized phase III study

**DOI:** 10.1186/1471-2407-14-155

**Published:** 2014-03-05

**Authors:** Toru Aoyama, Takaki Yoshikawa, Satoshi Morita, Junya Shirai, Hirohito Fujikawa, Kenichi Iwasaki, Tsutomu Hayashi, Takashi Ogata, Haruhiko Cho, Norio Yukawa, Takashi Oshima, Yasushi Rino, Munetaka Masuda, Akira Tsuburaya

**Affiliations:** 1 Department of Gastrointestinal Surgery, Kanagawa Cancer Center, 1-1-2 Nakao, Asahi-ku, Yokohama 241-0815, Japan; 2 Department of Biostatistics and Epidemiology, Yokohama City University Medical Center, Yokohama, Japan; 3 Department of Surgery, Yokohama City University, Yokohama, Japan

**Keywords:** Clinical trial design, Gastrointestinal surgery, Pathology

## Abstract

**Background:**

This randomized Phase III trial will evaluate whether the methylene blue-assisted technique is efficient for harvesting lymph nodes after radical surgery for gastric cancer.

**Methods/design:**

Patients that undergo distal or total gastrectomy with radical nodal dissection will be randomly assigned to Group A: the standard group, the lymph nodes (LNs) will be harvested from the fresh specimen immediately after surgery, or Group B: the methylene blue-assisted group, where the LNs will be harvested from specimens fixed with 10% buffered formalin with methylene blue for 48 hours after surgery. The primary endpoint is the ratio of the number of the harvested LNs per time (minute). The secondary endpoint is the number of harvested LNs. A 25% reduction in the ratio of harvested lymph-node/time (minute) was determined to be necessary for this test treatment, considering the balance between the cost and benefit. Retrospective data was used to estimate the ratio of the number of the harvested LNs per time (minute) to be 40/30 minutes in Group A. A 25% risk reduction and a rate of 40/22.5 minutes is expected in Group B. Therefore, the sample size required ensuring a two-sided alpha error of 5% and statistical power of 80% is 52 patients, with 26 patients per arm. The number of patients to be accrued was set at 60 in total, due to the likelihood of enrolling ineligible patients.

**Trial registration:**

UMIN000008624

## Background

Gastric cancer is the second most frequent cancer-related cause of death after lung cancer [1]. Surgical resection with radical lymphadenectomy is a standard treatment when gastric cancer is a local disease. Treatment strategy is determined based on the pathological diagnoses of tumor invasion and lymph-node metastasis. When determining N factor, TNM classification recommends nodal examination at least 16 or more regional lymph nodes (LNs) [2].

Nodal sampling from the specimens could be affected by physician’s experience, extent of dissection, type of gastrectomy, and the method for harvesting LNs. Surgeons harvest LNs from the fresh specimens immediately after surgery in Japan, while the pathologists from the specimens fixed with formalin after surgery in other countries [3]. Although surgeons may be more enthusiastic for harvesting LNs than pathologists, it must be tough to do such work just after surgery. On the other hand, nodal sampling may be difficult for pathologists who are not familiar with surgical anatomy. Moreover, the tissues fixed by formalin are hard and difficult to be separated; by which nodal sampling must be time-consuming work for most pathologists.

Methylene blue-assisted technique is another approach for harvesting LNs. The specimens are fixed with formalin with methylene blue after surgery, by which physicians are easy to pick up LNs stained by blue dye. Recently, a pathologist, Märkl reported in colon cancer that the methylene blue-assisted technique was significantly superior than the conventional methods in the number of and the time for harvesting LNs when the samples were fixed with formalin [4]. In gastric cancer, a few Japanese surgeons reported efficacy of this method in single arm studies [5,6]. It remains unclear whether the methylene blue-assisted technique is superior to standard method using the fresh samples.

Based on these, we conducted a randomized phase III study to confirm the superiority of the methylene blue-assisted technique compared with the standard approach using the fresh sample in harvesting LNs by surgeons after gastrectomy with radical lymphadenectomy for gastric cancer.

## Methods/design

### Purpose

The purpose of the study is to confirm the efficiency of the methylene blue-assisted technique in comparison to the standard approach using fresh samples for harvesting LNs by surgeons after gastrectomy with radical lymphadenectomy for gastric cancer.

### Study setting and protocol review

The study is an open-label, randomized Phase III trial. The protocol has been approved by the Institutional Review Committee of Kanagawa Cancer Center.

### Resources

Research grants are from the Kanagawa Standard Anti-cancer Therapy Support System (non-profit organization KSATTS).

### Endpoints

The primary endpoint is the ratio of the number of the harvested LNs per time (minute). The secondary endpoint is the number of harvested LNs. We set the ratio of the number of the harvested LNs per time (minute) as the primary endpoint because this is a measure for the efficacy; however, the stage migration is a measure for the accuracy not for the efficacy. Theoretically, the test arm has a superior in the efficacy but the same accuracy as compared with the control arm. To guarantee this, we set the stage migration as a secondary endpoint.

### Eligibility criteria

The tumors will be staged according to Japanese classification of gastric carcinoma 3rd English edition [7]. The inclusion criteria are:

(i). Histologically proven adenocarcinoma of the stomach.

(ii). Clinical stage 1–3 disease.

(iii). R0 resection is achieved by gastrectomy with D1+ or D2 lymphadenectomy according to Japanese gastric cancer treatment guidelines 2010 (ver. 3) as a primary treatment [8].

The patients that receive any other treatment before surgery will be excluded.

All participants gave written informed consent before study entry.

### Registration

Surgeons will register the eligibility criteria to the data center after confirmation during the surgery. The patients will be randomized and assigned to the standard group and the methylene blue-assisted group by a centralized dynamic method using the following factors: lymphadenectomy (D1+/D2), type of gastrectomy (subtotal/total), and surgical experience (less than 15 years/15 years-). The accrual was started in August 2012 and is to continue for 1 year.

#### Qualification of the participating surgeons

All surgeons have previous experience in harvesting LNs from more than 50 specimens.

## Methods

Patients with gastric cancer will undergo distal or total gastrectomy with radical lymphadenectomy. The extent of dissection will principally follow the third edition of the Gastric Cancer Treatment Guideline published by the Japanese Gastric Cancer Association [6]. Spleen-preserving D2 total gastrectomy is permitted in this study. The LNs will be harvested from the specimen immediately after surgery in the standard group. The specimens will be fixed with 10% buffered formalin with methylene blue in the methylene blue-assisted group, and the LNs will be harvested from the specimens. In this study, the concentration of the formalin is 10% in both arms. More, the concentration of the formalin is standardized throughout the study. All specimens were then fixed in 10 percent buffered formalin over two nights. LN harvesting in both arms are done by surgeons. The time for harvesting LNs is defined as that from the initiation to the termination. The ratio of the number of the harvested LNs per time (minute) is calculated.

### Statistical methods

The present study is a randomized phase III study to evaluate the efficiency of the methylene blue-assisted lymph node technique for harvesting LNs after surgery for gastric cancer. The primary endpoint is the ratio of the number of the harvested LNs per time (minute). A 25% reduction in the ratio of harvested lymph-node/time (minute) is necessary for this test treatment, considering the balance between the cost and benefit. Retrospective data from this institution was used to estimate the ratio of the number of the harvested LNs per time (minute) to be 40/30 minutes in the control arm. A 25% risk reduction in the test arm is expected, with a rate of 40/22.5 minutes in the test arm. This situation, will require a sample size of 52 patients, with 26 patients per arm to ensure a two-sided alpha error of 5% and statistical power of 80%. A total of 60 patients will be accrued due to the likelihood of enrolling ineligible patients.

The primary end point of the study is analyzed in the intent-to-treat (ITT) population using a Wilcoxon rank-sum test. The secondary endpoints, number of harvest lymph node and time of harvested lymph nodes are similarly analyzed using a Wilcoxon rank-sum test. Multivariate analysis is also used to analyze those endpoints with adjustment for clinically important background factors.

## Discussion

According to the results of the JCOG9501 trial, which was a Japanese multicenter phase III study to compare D2 and D2 plus para-aortic dissection for gastric cancer surgery, the median number of harvested LNs was 54 (range: 14–161) in the D2 group and 74 (range: 30–235) in the extended para-aortic dissection group [9]. In this trial, the experienced surgeons harvested LNs from the fresh specimens immediately after surgery. These data suggested that the number of harvested LNs tended to be much higher for extended dissection than normal D2, but that there was a wide variation even in D2 dissection. The first possible reason for this wide variation is that the number of LNs is different in each patient. However, no literature supports this possibility. The second possible reason is the difference in the gastrectomy type. The extent of nodal dissection was limited in the distal D2 gastrectomy compared with total D2 gastrectomy. The third possible difference is the skill of the surgeon for harvesting the LNs. In the present trial, we set the type of gastrectomy, extent of lymph node dissection, and surgeon’s experience as the stratification factors. Therefore, we will be able to minimize these effects regarding the differences in the standard method and methylene blue-assisted technique.

The hypothesis of the present study is that the methylene blue-assisted technique is superior to the conventional method, and is associated with a 25% reduction in the ratio of harvested lymph nodes/time (minutes). In general, the statistical hypothesis is determined by considering the balance between the risks and benefits of the standard arm and the test arm. However, there is no risk associated with the methylene blue-assisted technique for the patients. Therefore, it is possible to conclude that the methylene blue-assisted technique is effective even when the difference in the ratio of harvested lymph nodes/time is very small. However, it is not practical to statistically prove the significance of small differences. Considering the practical and acceptable sample sizes based on the previous data, we determined our statistical hypothesis.

In this trial, the primary endpoint is the ratio of the number of harvested LNs per time period (minutes). Our goal is to evaluate the efficacy of harvesting LNs by surgeons. On the other hand, the difference in the number of harvested LNs could produce stage migration between the two groups even though the number of nodal examinations was more than 16 in each group. Therefore, an expletory analysis is needed to evaluate the stage migration and cancer survival in the two groups.

## Competing interests

The authors declare that they have no competing interests.

## Authors’ contributions

TA, TY, JS, HF, KI, TH, TOgata, HC, NY, TOshima, YR, and AT have made substantial contributions to conception and design, or acquisition of data, or analysis and interpretation of data; TA, TY, SM, MM have been involved in drafting the manuscript or revising it critically for important intellectual content; and All authors have given final approval of the version to be published.

## Pre-publication history

The pre-publication history for this paper can be accessed here:

http://www.biomedcentral.com/1471-2407/14/155/prepub
